# Biological processes, properties and molecular wiring diagrams of candidate low-penetrance breast cancer susceptibility genes

**DOI:** 10.1186/1755-8794-1-62

**Published:** 2008-12-18

**Authors:** Núria Bonifaci, Antoni Berenguer, Javier Díez, Oscar Reina, Ignacio Medina, Joaquín Dopazo, Víctor Moreno, Miguel Angel Pujana

**Affiliations:** 1 Bioinformatics and Biostatistics Unit, and Translational Research Laboratory, Catalan Institute of Oncology, Bellvitge Biomedical Research Institute (IDIBELL), L'Hospitalet, Barcelona, Spain; 2 Unit of Infections and Cancer, Biomedical Research Centre Network for Epidemiology and Public Health (CIBERESP), Cancer Epidemiology Research Program, Catalan Institute of Oncology, Bellvitge Biomedical Research Institute (IDIBELL), L'Hospitalet, Barcelona, Spain; 3 Department of Bioinformatics, Functional Genomics Node and Biomedical Research Centre Network for Rare Diseases (CIBERER), Centro de Investigación Príncipe Felipe (CIPF), Valencia, Spain

## Abstract

**Background:**

Recent advances in whole-genome association studies (WGASs) for human cancer risk are beginning to provide the part lists of low-penetrance susceptibility genes. However, statistical analysis in these studies is complicated by the vast number of genetic variants examined and the weak effects observed, as a result of which constraints must be incorporated into the study design and analytical approach. In this scenario, biological attributes beyond the adjusted statistics generally receive little attention and, more importantly, the fundamental biological characteristics of low-penetrance susceptibility genes have yet to be determined.

**Methods:**

We applied an integrative approach for identifying candidate low-penetrance breast cancer susceptibility genes, their characteristics and molecular networks through the analysis of diverse sources of biological evidence.

**Results:**

First, examination of the distribution of Gene Ontology terms in ordered WGAS results identified asymmetrical distribution of Cell Communication and Cell Death processes linked to risk. Second, analysis of 11 different types of molecular or functional relationships in genomic and proteomic data sets defined the "omic" properties of candidate genes: i/ differential expression in tumors relative to normal tissue; ii/ somatic genomic copy number changes correlating with gene expression levels; iii/ differentially expressed across age at diagnosis; and iv/ expression changes after *BRCA1 *perturbation. Finally, network modeling of the effects of variants on germline gene expression showed higher connectivity than expected by chance between novel candidates and with known susceptibility genes, which supports functional relationships and provides mechanistic hypotheses of risk.

**Conclusion:**

This study proposes that cell communication and cell death are major biological processes perturbed in risk of breast cancer conferred by low-penetrance variants, and defines the common omic properties, molecular interactions and possible functional effects of candidate genes and proteins.

## Background

Technical and methodological advances in genome-wide assessment of genetic variation have provided tools for detecting low-penetrance susceptibility genes for common human diseases [1]. As a result of this progress, the last year has seen a spectacular increase in the number of published studies in which these types of variants or single nucleotide polymorphisms (SNPs) are detected. Projects such as the National Cancer Institute's Cancer Genetic Markers of Susceptibility (CGEMS) and work carried out by deCODE Genetics and the Breast Cancer Association Consortium have produced partial lists of the risk variants of different cancer types in diverse populations [2-4].

Whole-genome association studies (WGAS) are unbiased, which is highlighted by the fact that they identify unexpected candidate genes that are not strictly involved in *a priori *biological process such as DNA damage response in breast cancer [2-4]. The absence of bias is further revealed by the identification of possible master susceptibility loci for different cancer types, such as the convergence of risk variants at chromosome 8q24 [3,5-12]. The drawback of the agnostic nature of WGAS is the challenging statistical analysis and, thus, the biological interpretation of the results beyond single candidate SNPs and their *P *values. The vast number of variants interrogated means that *P *values below 10^-7 ^must be obtained to pass multiple-comparison corrections. Consequently, the number of samples needed to obtain the necessary statistical power is an important limitation, as is the fact that uncontrolled population stratification may introduce false positives. In addition, most variants seem to confer very modest risks in the order of 1.2–1.6 fold, which are hard to detect given the statistical difficulties described above. Indeed, current WGAS results contain thousands of SNPs and, by extension, thousands of candidate genes with unadjusted *P *values of < 0.05. As a result of these complications, the findings cannot be considered true positives until they have been replicated in an independent, preferentially larger-scale study [13,14].

Given these statistical constraints, possible biological interpretations of WGAS results are generally overlooked. In most cases genes are interpreted individually, and a gene ranked below the significance threshold will not be measured or experimentally characterized in relation to the disease or to genes that passed the threshold unless strong evidence is obtained from additional association studies. In this scenario, the fundamental principles of low-penetrance susceptibility genes and/or proteins (genes/proteins) – such as biological processes or pathways, properties and the molecular networks in which they commonly participate – have yet to be defined.

Systems-based interpretation of biological data is a common strategy in many areas of research [15-17]. It is clear that genes and proteins are organized in higher-order structures within complex molecular networks to execute biological functions [18]. The genes/proteins organized in these structures are the indivisible elements that are disrupted or regulated abnormally in disease but alterations of different genes/proteins in the same functional unit often converge in a common disease phenotype [19]. Genetic variability that confers risk of common diseases is also likely to converge at some level in specific processes or functions. Pioneering work by Wang and Bucan [20] has shown that the use of biological labels and microarray data analysis tools can facilitate the interpretation and priorization of candidate genes in WGAS.

Taking breast cancer as a model, we applied an integrative approach for uncovering the biological processes underlying breast cancer susceptibility mediated by low-penetrant alleles, as well as the genes/proteins and their properties and molecular interactions that are critical in cancer risk. Our strategy avoids the statistical constraints of WGAS by providing a method for prioritizing candidate markers based on the identification of common biological processes and characteristics. In addition, we provide hypotheses on the possible molecular mechanisms of risk between novel candidates and known susceptibility genes/proteins.

## Methods

### WGAS ordered gene lists

The breast cancer pre-computed WGAS data set released by the CGEMS initiative was downloaded from the corresponding public web site on September 2007. To examine biological information in WGAS results, we generated two complementary gene ranks: one according to the lowest *P *value per gene for the genotypic test in a genomic region of +/- 10 kilo bases (kb) at each locus, adjusted for age and hormone therapy [2]; and the other according to the lowest *P *value but also taking into account the direction of the association using the OR of the minor allele homozygotes (ORs of either > 1 or < 1). Assigned SNPs were curated using Ensembl gene annotations. Note that *P *values and ORs are not strictly comparable as they reflect different statistical analyses; the *P *values indicate the significance of an SNP in a logistic regression model, whereas the OR compares the magnitude of association of an allele against major homozygotes. The "one SNP-one gene" simplification was applied to obtain a single representation of each gene in the ranks. This approach might over-estimate large gene loci, and other strategies that account for the number of SNPs per gene, their linkage disequilibrium and allele frequencies could be used to enhance this analysis. The rank based on *P *values was then examined for differential representation of biological processes at one tail (low *P *values), while the rank based on ORs may differentiate disease-risk mechanisms (OR > 1) from protection mechanisms (OR < 1). By assigning SNPs as described above, a rank of 24,458 unique gene symbols (NCBI build 36.1) was obtained from an initial number of 528,173 SNPs [2]. Note that with *P *values of < 0.05, the original data set contains 26,859 SNPs corresponding to 7,611 genes. The number of unique genes in the OR-based rank was slightly lower (*n *= 24,135) because some of the SNPs had no data for minor homozygotes. The reference unit in our analyses was either the Entrez gene symbol or the Ensembl identifier (release 49), and other identifiers were converted to these references using BioMart [21]. Inconsistencies or missing values between Entrez and Ensembl identifiers were curated manually.

### GO term annotations

The Gene Ontology (GO) [22] annotations were downloaded from Open Biological Ontologies version 1.2, release 200804 (MySQL version). GO terms were assigned to gene symbols after record linkage in which regular expression searches were required. Splicing variants were collapsed for each gene symbol. Genes annotated at Level 4 or lower in the GO hierarchy were assigned to a parent in Level 3, but those also occurring at Level 2 were excluded. This analysis gave 14,659 (~60%) genes annotated (271 terms and a median of 641 genes in each term) from the starting list of 31,591 while 24,458 of the genes were present in the WGAS, of which 11,675 were annotated. The remaining ~40% of genes were unannotated, mainly because they represent uncharacterized genes/proteins or do not contain known biological features. The same procedure was used when evaluating terms at Level 4 giving 1,867 gene sets.

### Analysis of rank partitions

We implemented the procedure devised by Al-Shahrour and colleagues [23,24] to examine outputs flexibly (Additional file 1). The implementation was performed in the R language and environment [25] and consisted of the following steps, as defined by the original authors: 1/ the list of gene/protein identifiers was ordered according to a measure of association; 2/ a selected number of partitions *p *was applied, each of which separated the ordered list into two parts, and used the index in order to force each partition to increase with the same number of genes (we show results for 50 partitions, but we also explored the range between 30 and 50 that was recommended in the original publication [24], which revealed similar results); 3/ for each partition, the frequencies of genes/proteins with a specific GO term annotation were compared using a Fisher's exact test for two-by-two contingency tables; 4/ the previous step was repeated for *m *terms; 5/ a multi-testing adjustment procedure was applied to *P *values taking into account *p *× *m *tests, using the FDR approach [26] implemented in the *multtest *package [27]; 6/ significant terms were selected and graphics were created in R. In comparison with GSEA, the partitions methodology may be capable of detecting modest differences [24], although it is probably less effective at providing detailed interpretations of the position of these differences. One hundred permutations of gene order in WGAS ranks were examined for possible asymmetries obtained by chance. In addition, in our analyses using partitions, we controlled for possible background bias of annotated and unannotated genes for any term.

### GSEA analysis

The GSEA algorithm was applied using the Java implementation [28], with ordered gene lists and annotations from Level 3 and 4 Biological Process GO terms, and the enrichment weighting exponent *p *= 1 (except when examining gene index ranks). The statistical significance (nominal *P *value) of the enrichment score (ES) was calculated in the implementation by permuting the class labels (genes) 1,000 times. Log-transformed *P *values were used in the analysis of WGAS-ordered gene lists.

### Analysis of breast cancer-related data sets

Differential expression between normal breast tissue and tumors was assessed at the genome-wide level using the data set provided by Richardson and colleagues [29]. Differences were evaluated using the *t*-statistic across all tumors and also for basal-like or non-basal-like subclasses. No differences were observed in GO term profiles so we used the comparison with all tumors. Genetic alterations in tumor subclasses were evaluated using copy number information from the study of Chin and colleagues [30]. For each SNP-gene position of the WGAS an average copy number was obtained in each tumor class. To calculate correlations between gene expression and copy numbers, we first obtained average gene expression values in tumor classes using all possible probes mapping each gene, and then calculated correlations with copy numbers using the Pearson correlation coefficient (PCC). To evaluate prognosis we used the data set of Chang and colleagues [31], which contains 295 breast tumors. We fitted a Cox regression model to each probe using disease-free survival time information. Models were fitted adjusting for ER tumor status and grade, and likelihood ratio tests were calculated to evaluate the effect of microarray probe values on survival. Genes were then ordered according to hazard ratios and/or *P *values using only the extreme probe results. To evaluate age at diagnosis we used the same data set and fitted a linear model for each probe, adjusting for ER tumor status and grade. Next, we applied the same procedure as that used for the prognosis analysis to obtain a definitive ordered list of genes based on the regression coefficient and the corresponding *P *values. The same data set was used to assess expression differences between ER-negative and ER-positive breast tumors and for co-expression analyses with benchmark breast cancer genes using the PCC. In addition, we investigated expression perturbations after *BRCA1 *depletion in MCF10 cells [32], using fold-changes, and expression perturbations between BRCA1 and sporadic breast tumors (non-hereditary ER-negative and grade 3) using the *t*-test [33]. Finally, we examined gene expression changes in tissue abnormalities precursors of breast cancer, using the *t*-test [34].

### Analysis of the human interactome network

The human interactome network was built by combining three previously published data sets, which consist mainly of experimentally verified interactions. The data set based on the Human Protein Reference Database (HPRD) was combined with high-confidence yeast two-hybrid interactions from Rual and colleagues [35] and Stelzl and colleagues [36]. Orthology-based predictions and homodimers were excluded from our analyses. Shortest paths were calculated using only the giant network component and the geodesic formulation given by Freeman [37] using the R programming language [25]. GO term annotations were used as detailed above. Proportions of annotations in direct and one-hop interactors of benchmarks were evaluated in the giant network component using as controls seed proteins annotated with the same terms as the benchmark that was being compared. *P *values were then computed using empirical distributions.

### Genetics of gene expression

The Dixon and colleagues data set [38] was down-loaded from the public web site and analyzed focusing on SNPs with lod scores of > 2.3. Variants at r^2 ^> 0.8 were identified using Phase II HapMap release 21a data for individuals with European ancestry. Data is provided for lod scores of > 6 and SNPs-genes in the combined rank, whereas information for variants at lod scores of > 2.3 and r^2 ^> 0.8 is available from the authors. To avoid any bias, the network and simulations only refer to the original SNPs annotated by Dixon and colleagues [38] and exclude variants at r^2 ^> 0.8. Networks were generated in Cytoscape [39] and using the R programming language [25]. SNPs at each gene locus (+/- 10 kb) were collapsed into a single node for network representation.

## Results

### Biological processes in breast cancer risk

**Figure 1:**
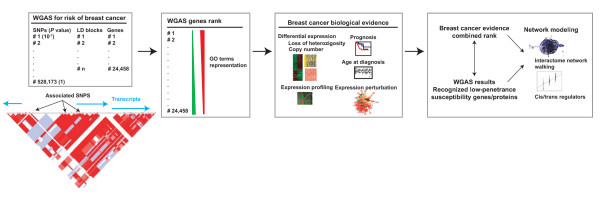
**Strategy for candidate gene prioritization in WGAS results**. Given a WGAS such as the breast cancer study of the CGEMS initiative [2], ~500,000 SNPs were initially interrogated, which represent a lower number of linkage disequilibrium (LD) blocks in which 24,458 known human genes are distributed. Even when a clear LD block contains several significant SNPs, different genes may be present and molecular and/or functional analyses are required to determine the most likely candidates and their interactions. To obtain this information at the genome-wide level, we propose first to use GO terms to examine the WGAS rank for asymmetries in biological processes. These asymmetries will then be used to guide the analysis of omic data sets relevant to breast cancer biology. Next, higher-level data analyses – protein-protein interactions that may be over-represented for the same processes, and variants in cis/trans affecting germline gene expression levels that lead to hypotheses on the possible functional effects of risk alleles – are performed using a combination of evidences, WGAS results and recognized low-penetrance susceptibility genes/proteins or benchmarks.

Breast cancer is probably the paradigm of deeply characterized neoplastic process at many molecular levels. The key to this study was the public availability of the landmark WGAS for breast cancer risk released by the CGEMS initiative [2]. We analyzed the results of this WGAS alongside various omic data sets of breast cancer and normal cellular conditions, following a biology-driven strategy based on the asymmetrical representation of biological information in ordered gene lists (Figure 1). The combined rank provides a prioritized list of gene/protein candidates and their interactions in pathology.

To examine the distribution of biological information in WGAS ordered gene lists (see Methods), we compiled Level 3 Biological Process GO term annotations and applied two complementary algorithms: one that uses the "partitions" concept devised by Al-Shahrour and colleagues [23,24] (the implementation of this algorithm is available in Additional file 1); and the Gene Set Enrichment Analysis (GSEA), which evaluates asymmetries based on the Kolmogorov-Smirnov statistic [40]. The first algorithm generates *p *partitions in an ordered gene list and then computes a Fisher's exact test for each of the *p *two-by-two contingency tables to detect asymmetries between the top and the bottom parts of the list. Next, *P *values are corrected based on the false FDR approach [26]. All known genes in the human genome NCBI build 36.1 were included in the examination of WGAS ranks. In our implementation we took into account both annotated and unannotated genes/proteins, which we found to prevent false positives due to background asymmetrical distributions (not shown).

**Figure 2:**
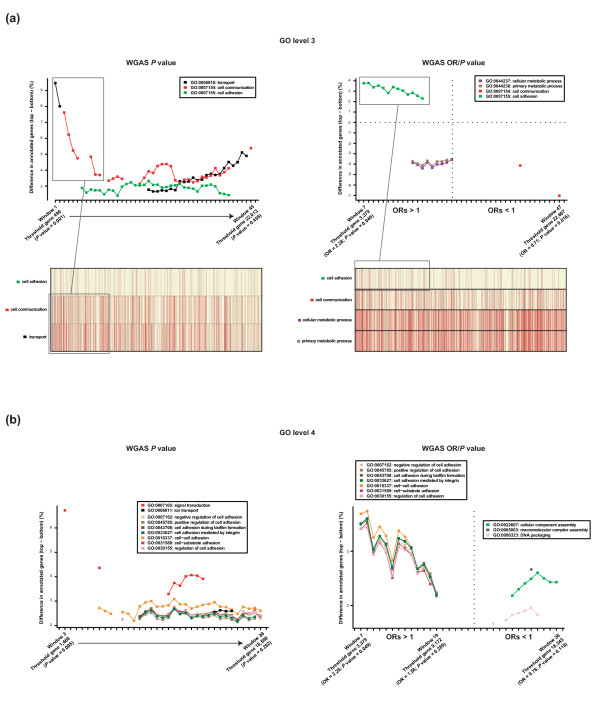
**WGAS rank asymmetries for specific biological processes**. **(a) **Graphical representation of over- and/or under-representation of biological processes in partitions of WGAS ranks using Level 3 GO annotations. Top left panel, results of the analysis of the WGAS rank according to the lowest *P *value per gene locus. Differences are always shown from top to bottom, so the top shows over-representation in the GO terms Transport and Cell Communication. Graphics show significant partitions. Bottom left panel, graphical representation of the positions of genes annotated with GO terms distributed asymmetrically in the WGAS *P *value rank. Right panels, results of the analysis of the WGAS rank according to ORs and to *P *values. This analysis seems to better capture the differences in risk (ORs > 1) associated with the over-representation of Cell Adhesion. Under-representation (negative differences when comparing top with bottom parts) of Metabolic processes annotations is also suggested with ORs of > 1. The graphical representation of gene positions shows clear differences between Cell Adhesion and more complex patterns – perhaps with different gene subgroups – for Cell Communication and Metabolic processes. **(b) **Graphical representation of over- and/or under-representation of biological processes in partitions of WGAS ranks using Level 4 GO annotations. Left panel, child terms of Cell Communication (Signal Transduction), Transport (Ion Transport) and Cell Adhesion (rest of terms shown in the inset) are over-represented at *P *values of up to 0.262. Right panel, over-representations in the WGAS OR/*P *value ordered list as shown in the insets.

Of the 271 terms in Level 3, asymmetries were identified in the distribution of Transport, Cell Communication and Cell Adhesion processes using the partitions methodology and two possible WGAS ranks (Figure 2a and Methods). To evaluate the significance of these results we performed the same analysis for 100 permutations of gene order. None of the permutations showed significant differences for any of the 271 terms at any partition. In addition, when the GSEA algorithm and our Level 3 annotations were used, the greatest asymmetries were found in the same terms (particularly Cell Adhesion), and smaller differences were observed in other terms including Cell Development and Death (Additional file 2). The consistency of the results suggests that the terms identified represent key biological processes in breast cancer risk conferred by common variants.

As expected, profile differences were observed between the two defined WGAS ranks, and Cell Adhesion was more clearly asymmetrically distributed in the ordered gene list that takes into account the lowest *P *value per gene locus and the corresponding odds ratio (OR) (Figure 2a, right panels). Cell Communication is visibly asymmetrically distributed in the *P *value based rank, whereas the inclusion of OR criteria suggests the existence of gene subgroups in this process associated with risk. Under-representation of genes involved in Metabolism was also revealed at the top of the rank, which leads us to speculate that common variants in this process play a protective role.

### Fine mapping of processes

Given the asymmetries at Level 3, and taking into account that the gene sets were relatively large, candidate processes were narrowed down using child terms at Level 4. In agreement with results above, terms for Transport, Cell Communication and Cell Adhesion were found to be distributed asymmetrically in both WGAS ranks (Figure 2b). For example, Signal Transduction was a child of Cell Communication and was found to be over-represented at low *P *values. Several recognized low-penetrance susceptibility genes are annotated in this term (*AURKA *[41-45],*CASP8 *[46],*LSP1 *[3]and *TGFBR1 *[47-51]). The child terms for Transport and, in particular, for Cell Adhesion also showed similar asymmetries to those at Level 3 (Figure 2b). Profiles were also found to be consistent with the list ordered by OR/*P *value, with many child terms for Cell Adhesion over-represented at ORs of > 1. These observations corroborate the identification of key processes – in particular Cell Communication and Cell Adhesion – mediating breast cancer risk.

### Breast cancer-related properties

To further define the characteristics of candidate susceptibility genes in breast cancer conditions, we examined various sources of biological evidence according to the observed WGAS rank GO asymmetries. Nine types of evidence were examined (Additional file 3):

1/ Differential expression between normal breast tissue and tumors [29] (accounting for different known molecular classes of breast tumors [52]).

2/ Differential expression between normal breast tissue at terminal duct lobular units and hyperplasic units [34].

3/ Correlations between transcript profiles using as benchmarks known genes of low/moderate risk (*ATM, AURKA, BRIP1, CASP8, CHEK2, FGFR2, HMMR, LSP1, MAP3K1, PALB2, RASSF1, TGFBR1 and TNRC9*), high risk (*BRCA1 *and *BRCA2*) and cancer syndromes (*LKB1, PTEN *and *TP53*) [53].

4/ Somatic loss of heterozygosity and copy number alterations in tumors [30] (accounting for the different known tumor types).

5/ The correlation between somatic copy number alterations and transcript profiles [30] (again, accounting for the different known tumor types).

6/ The dependence of the estrogen receptor (ER) pathway signaling on differential expression between ER-positive and ER-negative tumors [31,33,54].

7/ The association between gene expression and patient prognosis [31,33,54] (adjusting for major confounding variables of ER status and tumor grade).

8/ The association between gene expression in tumors and patient age at diagnosis [31,33,54] (again, adjusting for major confounding variables of ER status and tumor grade).

9/ Expression perturbation in BRCA1 tumors (tumors originating in carriers of germline *BRCA1 *mutations) relative to sporadic (non-hereditary) tumors [33], or after depletion of *BRCA1 *in a non-tumorigenic cell model [32,55].

These different types of evidence characterize different aspects of breast cancer biology, including the following: the identification of putative tumor suppressors and oncogenes by analyzing differential expression and/or somatic genetic alterations [30]; genes with a role in the early stages of breast tissue transformation [34]; hormone dependencies that may be related to susceptibility, as noted recently for newly identified low-penetrance susceptibility genes [4]; expression perturbations in BRCA1 tumors that may reveal functional relationships with high-penetrance genes/proteins [32,56,57]; and associations with age at diagnosis that may also indicate critical molecular roles in initiating tumorigenesis [57].

**Figure 3:**
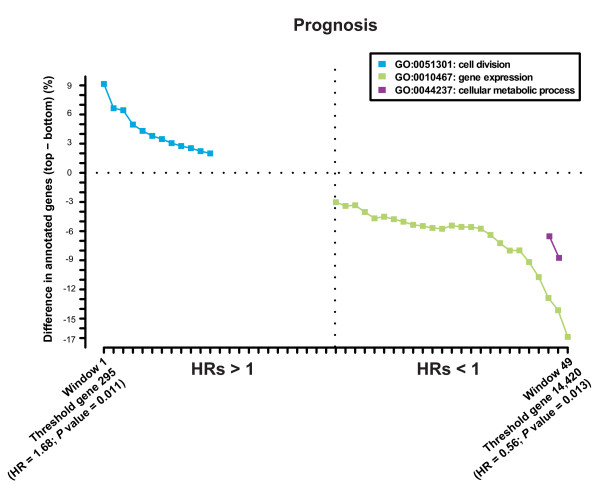
**Asymmetries in biological evidence of breast cancer**. An example of the results of applying the methodology used to examine the WGAS ranks to a breast cancer biological evidence data set. This analysis identifies the association between gene expression levels and patient survival or prognosis measured by the hazard ratio (HR). The results suggest an association between poor prognosis (HRs > 1) and genes involved in Cell Division, and between good prognosis (HRs < 1) and Gene Expression and Metabolic processes. Importantly, the association between cell proliferation and poor prognosis has been demonstrated in previous studies using different approaches [58-63].

Analysis of the evidence described above identified biological processes consistent with existing knowledge in the literature. For example, Cell Division was distributed asymmetrically in genes ranked according to the hazard ratio that measures survival probability (Figure 3), which is consistent with the fact that the potential for cell proliferation can be considered a strong predictor of prognosis or metastasis [58-63].

**Figure 4:**
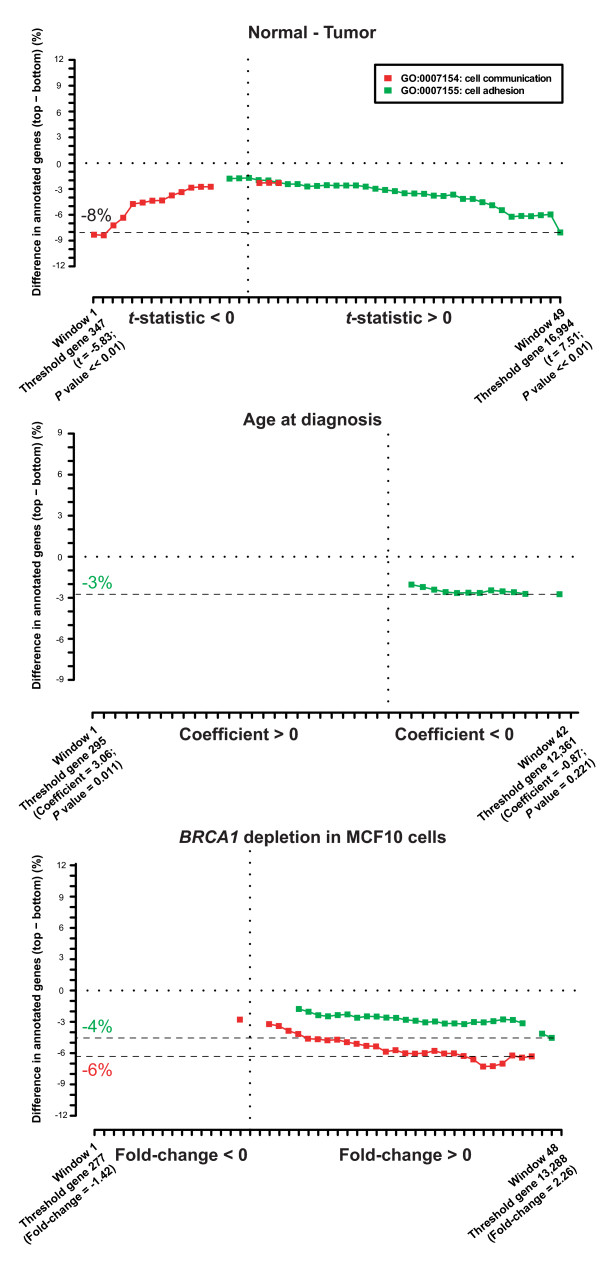
**Asymmetries for Cell Communication and Cell Adhesion**. Of the biological evidence of breast cancer examined in this study, three cases showed asymmetries in biological processes that are similar to those observed in the WGAS ranks. Three cases showed similar asymmetries for Cell Adhesion: 1/ top panel, differential expression between normal breast tissues and tumors measured using the *t*-statistic, as a result of which genes involved in Cell Adhesion are over-represented at the bottom, which indicates that they are generally under-expressed in tumors, while Cell Communication is under-represented at the top (note that both patterns follow the same direction); 2/ middle panel, association between age at diagnosis and gene expression levels measured using the coefficient from the linear model, so coefficients < 0 indicate association with early age at diagnosis, which is consistent with the expected contribution of genetic effects to breast cancer risk [57]; 3/ bottom panel, fold change in gene expression changes between *BRCA1*-depleted and control-treated MCF10A cells, which indicates possible molecular and/or functional dependencies on processes linked to breast cancer risk [57]. Differences in annotation percentages between top and bottom range from -8% to -4% for the most significant partition at the bottom end. On the basis of these results, all three ranks were inverted and combined for comparison with the WGAS results.

Of the nine types of evidence described above, three showed similar asymmetries in Cell Adhesion to those observed in the WGAS ranks: differential expression between normal breast tissue and tumors, patient age at diagnosis, and *BRCA1 *depletion in MCF10A cells (comparison of BRCA1 and sporadic tumors also revealed similar asymmetries, but it was excluded from the analyses below to avoid duplication). Two of these data sets also showed similar asymmetries for Cell Communication (Figure 4). As mentioned above, permutation analysis of gene ranks did not show asymmetries in any process, which indicates that these evidences are useful for categorizing and defining the omic properties of genes contributing to breast cancer risk.

Asymmetries in these processes were also observed in tumor subclasses when the rank of correlations between somatic genomic alterations and gene expression levels were examined. This was found principally in luminal A tumors (Additional file 4), and although the corresponding combined rank did not vary considerably from those of the three types of evidence described above, it captured as likely candidate genes those involved in ER signaling such as *TFF1 *(Additional file 5), which was expected for a hormone-dependent tumor class [52]. This specific evidence for a given subclass can then be used when examining breast cancer subtypes.

### Evaluation of a combined evidence rank

Given that three breast cancer conditions showed similar asymmetries in processes to those observed in the WGAS ranks, a combined rank of these conditions might provide a prioritized list of more likely candidates. This analysis was performed using all genes in common between these three omic data sets (*n *= 8,986) and the final rank was created using the average position (Additional file 6). Although there is not a large "gold standard" of low-penetrance susceptibility genes, some features of the combined rank suggest that it is biologically meaningful in the assessment of genetic risk factors.

Examination of the 50 top-ranked genes in the combination identified candidate tumor suppressors and/or oncogenes from the literature (*DKK3 *[64] and *TFPI2 *[65]), genes with variants that confer breast cancer risk (*IGF1 *[66]) and, notably, four genes (*PDGFRA, PDGFRL, MAP3K12 *and *NTRK2*) whose products participate in the MAPK signaling pathway, where known susceptibility genes also participate (*FGFR2 *and *MAP3K1 *[2-4]) (Figure 7, shows the results for the 50 top-ranked genes in the combined evidence ranking ordered by their lowest WGAS *P *value). This 50-set also contains genes previously linked to breast cancer prognosis, metastasis or treatment response (*BCL2 *[67], *CXCL12 *[68-70] and *FBLN1 *[71,72]). In addition, consistent with predicted relationships in this set, experimental studies have demonstrated interactions between the corresponding proteins in neoplasia; for example ABTB1 and EGR2 are mediators of PTEN tumor suppressor function [73]. These observations support the hypothesis that the combined rank contains numerous functional and molecular associations of relevance for breast tumorigenesis.

**Table 1:**
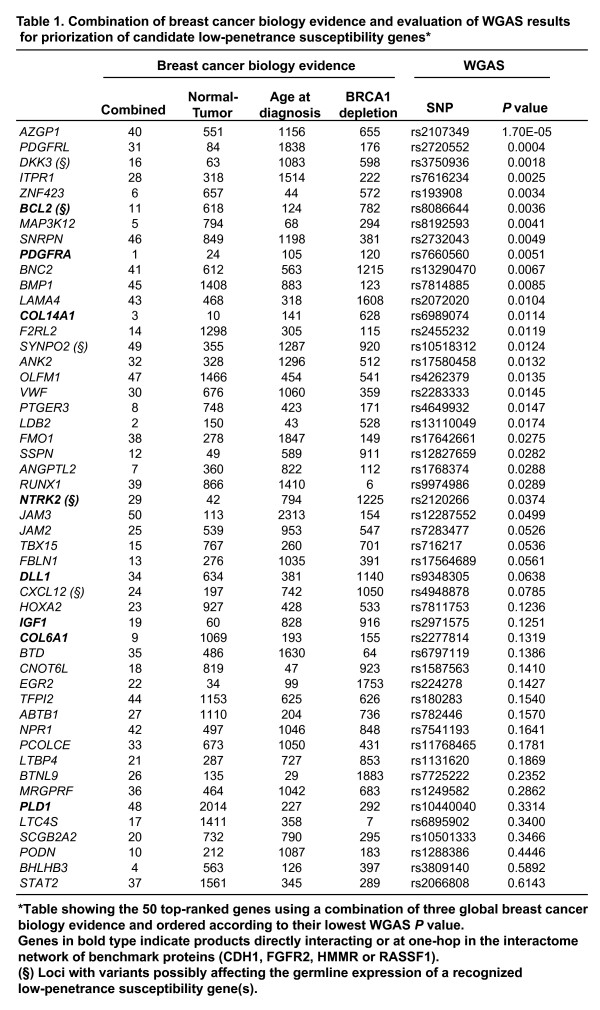
Combination of breast cancer biology evidence and evaluation of WGAS results for priorization of candidate low-penetrance susceptibility genes

The second position of the combined ranking that takes into account the WGAS results is occupied by the platelet-derived growth factor receptor-like (*PDGFRL*) gene, while the first gene in the combined rank is *PDGFRA *(Figure 7).* PDGFRA *is expressed in invasive carcinomas and is associated with aggressiveness [74], and, importantly, *PDGFRL *is mutated in cancer cells [75,76] and maps at chromosome 8p22-p21, where it is thought to map a breast cancer tumor suppressor gene(s) [77-79]. More recently, an integrative approach based on disease-specific pathways has revealed that *PDGFRL *may play a critical role in promoting breast tumorigenesis [80]. Our independent observations of breast cancer risk may lead to the replication of the WGAS findings for these *PDGFR *genes and others shown in Figure 7. In this way, evaluation of genes with somatic point mutations in breast tumors as compiled in the COSMIC database (release v36) [81] placed *MAP3K12 *at the top of the combined rank (Additional file 7), which reinforces the putative involvement of the MAPK signaling pathway and supports *MAP3K12 *as a likely candidate.

### Examination and integration of higher-order evidence

**Figure 5:**
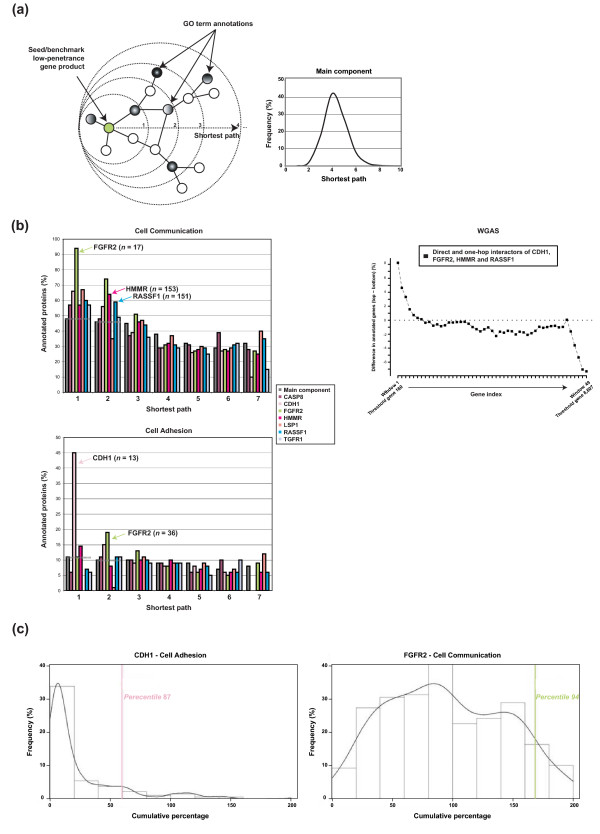
**Same biological processes in the interactome network neighborhoods**. **(a) **Left panel, strategy used to examine the interactome network; given a seed or benchmark protein encoded by a recognized low-penetrance susceptibility gene and using a shortest path algorithm, we calculated at each step the percentage of nodes annotated with Cell Communication or Cell Adhesion among proteins annotated with any term (excluding non-annotated proteins). Right panel, distribution of all possible short paths in the giant network component. **(b) **Left panels, results for percentages in short paths of up to seven steps for benchmark proteins. Over-representation in Cell Communication and Cell Adhesion annotations is suggested for CDH1, FGFR2, HMMR and RASSF1 at direct and/or one-hop interactions. Right panel, asymmetrical distribution of CDH1, FGFR2, HMMR and RASSF1 direct and one-hop interactors in the complete WGAS rank. **(c) **Over-representation of processes in the one-hop neighborhood of CDH1 or FGFR2 (vertical lines) using as controls seed proteins with the same Level 3 annotations (curves). The x-axis represents the cumulative percentage up to 200. The CDH1 and FGFR2 percentiles are shown.

Correlations across different biological levels provide better proof of molecular associations and their possible perturbation in disease [16,18,82]. We examined the network of protein-protein interactions (interactome network) of recognized low-penetrance susceptibility gene products (hereafter referred to as benchmarks) for proportions of annotations in Cell Communication and Cell Adhesion. Proportions of annotations were compared between interactors of benchmarks and the average in the giant network component and, to avoid bias, only proteins annotated at any GO level were considered. Using as network seeds those nodes representing seven benchmark proteins with at least one known interaction in the giant component (CASP8, CDH1, FGFR2, HMMR, LSP1, RASSF1 and TGFBR1), over-representation of Cell Communication and Cell Adhesion was detected in several neighborhoods using the shortest path measure, particularly in direct and one-hop interactors (Figure 5a/b). The benchmark neighborhoods showing the highest over-representation of these processes were those corresponding to CDH1, FGFR2, HMMR and RASSF1 (Additional file 8).

To assess which of these benchmarks shown the maximum information at the interactome level for breast cancer risk, we calculated the probability of showing similar proportions of annotations in the giant component and, to avoid functional bias, used as controls seed proteins with the same annotations at Level 3 as each of the benchmarks being compared. The results of this controlled analysis suggest higher enrichment of the processes in the direct or one-hop interactors of CDH1 and FGFR2 (percentile 87 and 94, respectively) (Figure 5c). This observation suggests the close interactors of these low-penetrance susceptibility gene products as more likely candidates.

The results in the interactome network provide additional information that can be combined discretely with the rank in Figure 7. Consequently, annotating this rank for direct and one-hop interactors of CDH1, FGFR2, HMMR and RASSF1 provides a more restricted list of likely candidates. Again, this set contains previously defined candidates such as IGF1 [66] and members of the MAPK signaling pathway such as NTRK2 and PDGFRA, which are found in the one-hop neighborhood of FGFR2.

### Functional effects of variants and their evaluation in the combined rank

To determine the possible functional effects of risk variants in candidates, we examined differences in germline expression levels correlating with genetic variation, using the data set of Dixon and colleagues [38] derived from lymphoblastoid cell lines. To search SNPs we used the original data or, in cases which provided no information for an SNP, variants at linkage disequilibrium r^2 ^> 0.8 according to HapMap individuals with European ancestry [83]. In this analysis we not only examined single SNP/gene effects (Additional file 9) but also generated expression-perturbation networks in which nodes are formed by gene loci and edges represent direct or indirect expression effects, possibly mediated by coding and/or regulatory SNPs in candidate genes (see Methods).

**Figure 6:**
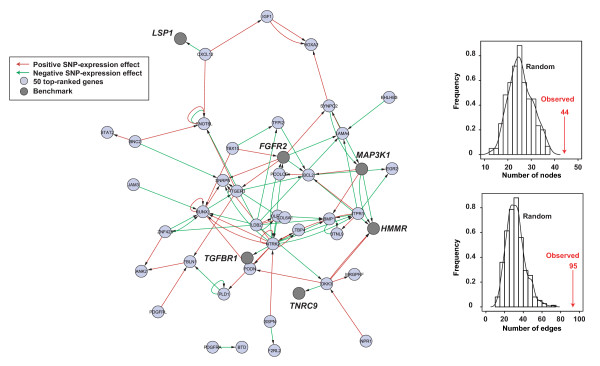
**Functional effects and associations between candidates and benchmarks**. Left panel, network of transcriptional perturbations mediated by SNPs at gene loci. Nodes represent SNP/gene loci of the 50 top-ranked candidates (Table 1) or of benchmarks, and edges represent the direction of the effect on gene expression, as shown in the inset. To avoid bias, we excluded those SNPs that are not annotated in the original data set of Dixon and colleagues [38]. Right panels, network results of the analysis of 100 randomly chosen sets of 50 genes and the same benchmarks (histograms and curves) compared to the observed values in the left panel (vertical arrows), for connected nodes (top) or edges (bottom).

Taking as candidates the 50 top-ranked genes from Figure 7, we identified many edges between their loci and with benchmarks (Figure 6, left panel). New candidates may then be prioritized based on their high centrality in the network (*BCL2*, *BMP1*, *NTRK2*, *PTGER3 *or *RUNX2*) or by the fact that they connect two benchmarks (*DKK3 *and *NTRK2 *connect *HMMR-TNRC9 *and *FGFR2-TGFBR1*, respectively), which suggests a possible risk effect through the expression perturbation of known low-penetrance susceptibility genes.

To evaluate the biological significance of this network, we performed similar analyses with 100 randomly chosen sets of 50 genes and the same benchmarks. The connectivity was higher for the 50 top-tanked genes in the combined rank than for any of the randomly generated networks, both for the number of nodes and the number of edges (Figure 6, right panels). This observation supports the functional association between the 50 top-ranked candidates and, importantly, the association with known genes of breast cancer risk. These results also provide many functional hypotheses of genetic variants in re-defined candidates that may influence breast cancer susceptibility. Overall, this integrative study identifies candidate low-penetrance breast cancer susceptibility genes and the corresponding wiring diagram of molecular interactions.

## Discussion

This study identifies biological processes that play key roles in breast cancer risk, which are revealed by asymmetrical distributions of GO terms in complete WGAS ranks. Common variants that affect, in particular, the function of genes/proteins in Cell Communication and Cell Adhesion probably confer breast cancer risk to a greater extent than variants in genes associated with different processes. Thus, this study provides a foundation for the analysis of fundamental issues in breast cancer risk conferred by low-penetrant alleles.

The involvement of Cell Communication and Cell Adhesion is intriguing given their long-known contribution to epithelial neoplasia, although typically at the somatic level [84]. Our results may link initial molecular perturbations to subsequent events in cancer progression, which suggests a more continuous path than previously thought between germline and somatic alterations. This hypothesis was highlighted primarily by the identification of risk variants at the *FGFR2 *and *MAP3K1 *loci – two genes known to be somatically altered in human cancer and whose products are involved in signal transduction among other processes [85,86]. These considerations apply to sporadic breast cancer but may also provide insights into the mechanisms of high-penetrance susceptibility genes since risk variants at low-penetrance loci also contribute to the risk of *BRCA1 *and *BRCA2 *mutation carriers [87]. Overall, these observations point to a molecular diagram for breast cancer risk that may be more complex than previously thought, probably based not only on the alteration of the DNA damage response.

However, the limitations of this study must also be presented. Firstly, methodological constraints might hamper the detection of subtle asymmetries of GO terms. To improve sensitivity, WGAS results could be ordered by combining the effect and magnitude of variants using Bayesian principles. Alternatively, different biological labels could be used – we considered annotations of pathways [88] that did not reveal significant differences (not shown). Secondly, although the application of the average across ordered lists detected genes/proteins known to be involved in breast tumorigenesis (Figure 7), more sophisticated methods for combining ranks could improve the detection of susceptibility genes. Finally, this study is limited by the analysis of a single WGAS data set with certain epidemiological specificities [2], thus any candidate highlighted here should be examined in an independent epidemiological study.

Based on the observations from the WGAS ranks, we then examined different breast cancer conditions that could provide further categorization of candidates and reveal the common properties of low-penetrance susceptibility genes. Variants of these genes appear to correlate with transcripts that are differentially expressed in tumors, with somatic copy number changes that correlate with gene expression, differentially expressed across age at diagnosis, and which show changes in expression level after depletion or in the presence of *BRCA1 *mutation. Correlations between somatic genomic alterations and gene expression may indicate tumor suppressors or oncogenes, depending on the direction of the correlation [89]. The association with age at diagnosis (identified when adjusting for confounding variables) supports a role in cancer risk, for example differential expression at early age [57]. Finally, changes mediated by *BRCA1 *perturbation suggest molecular or functional dependencies with high-penetrance susceptibility genes/proteins [56,57]. This study suggests that these are frequent features of low-penetrance breast cancer susceptibility genes.

Combination of these evidences provides a comprehensive rank to evaluate WGAS results beyond statistical constraints. This observation is supported by analyses at higher-order molecular levels. Direct and one-hop physical interactors of susceptibility benchmarks are over-represented in the same biological processes as the top of the WGAS ranks. In addition, modeling of a germline transcriptional regulatory network identifies connections with benchmarks but also reveals higher connectivity than randomly expected, which supports that these genes/proteins function in biologically related processes. We propose this integrative study provides the basis for better biological knowledge of the genes/proteins, their omic properties and interactions that mediate the initial steps of breast tumorigenesis. This strategy may be useful for revealing the genes/proteins and their wiring molecular diagrams of susceptibility for other cancer types where WGAS are being carrying out and have vast omic data sets.

## Conclusion

This study proposes biological criteria that may facilitate the prioritization of candidate genes in WGAS for breast cancer. The identification of the processes, omic properties and molecular interactions may represent the first step towards a more comprehensive understanding of the molecular mechanisms of risk of breast cancer conferred by of low-penetrance susceptibility genes.

## Competing interests

The authors declare that they have no competing interests.

## Authors' contributions

NB, AB participated in the study design and performed the WGAS and omic data analyses. OR performed the analysis of the regulatory network. JD and VM participated in scientific discussions and helped with the overall interpretation of the data. IM and JD helped in the analyses using the partition algorithm. MAP conceived and designed the study and drafted the manuscript. All authors read and approved the final manuscript.

## Pre-publication history

The pre-publication history for this paper can be accessed here:



## Supplementary Material

Additional file 1 Algorithm for partition strategy in studying ordered lists. Click here for file

Additional file 2 Results of the GSEA algorithm. Click here for file

Additional file 3 Analyses of breast cancer data sets. Click here for file

Additional file 4 Asymmetries in the rank of somatic copy number-gene expression correlations in luminal A tumors. Click here for file

Additional file 5 Combined rank of breast cancer biological evidence including somatic copy number-gene expression correlations in luminal A tumors. Click here for file

Additional file 6 Combined rank of common breast cancer biological evidence. Click here for file

Additional file 7 Combined rank of COSMIC genes. Click here for file

Additional file 8 Direct and one-hop interactors of benchmark low-penetrance susceptibility gene products. Click here for file

Additional file 9 SNPs with possible functional effects at lod score > 6. Information for variants at lod scores > 2.3 is available from the authors. Click here for file

## References

[B1] Kruglyak L (2008). The road to genome-wide association studies. Nat Rev Genet.

[B2] Hunter DJ, Kraft P, Jacobs KB, Cox DG, Yeager M, Hankinson SE, Wacholder S, Wang Z, Welch R, Hutchinson A, Wang J, Yu K, Chatterjee N, Orr N, Willett WC, Colditz GA, Ziegler RG, Berg CD, Buys SS, McCarty CA, Feigelson HS, Calle EE, Thun MJ, Hayes RB, Tucker M, Gerhard DS, Fraumeni JF, Hoover RN, Thomas G, Chanock SJ (2007). A genome-wide association study identifies alleles in *FGFR2 *associated with risk of sporadic postmenopausal breast cancer. Nat Genet.

[B3] Easton DF, Pooley KA, Dunning AM, Pharoah PDP, Thompson D, Ballinger DG, Struewing JP, Morrison J, Field H, Luben R, Wareham N, Ahmed S, Healey CS, Bowman R, Meyer KB, Haiman CA, Kolonel LK, Henderson BE, Le Marchand L, Brennan P, Sangrajrang S, Gaborieau V, Odefrey F, Shen C-Y, Wu P-E, Wang H-C, Eccles D, Evans DG, Peto J, Fletcher O (2007). Genome-wide association study identifies novel breast cancer susceptibility loci. Nature.

[B4] Stacey SN, Manolescu A, Sulem P, Thorlacius S, Gudjonsson SA, Jonsson GF, Jakobsdottir M, Bergthorsson JT, Gudmundsson J, Aben KK, Strobbe LJ, Swinkels DW, van Engelenburg KC, Henderson BE, Kolonel LN, Le Marchand L, Millastre E, Andres R, Saez B, Lambea J, Godino J, Polo E, Tres A, Picelli S, Rantala J, Margolin S, Jonsson T, Sigurdsson H, Jonsdottir T, Hrafnkelsson J (2008). Common variants on chromosome 5p12 confer susceptibility to estrogen receptor-positive breast cancer. Nat Genet.

[B5] Gudmundsson J, Sulem P, Manolescu A, Amundadottir LT, Gudbjartsson D, Helgason A, Rafnar T, Bergthorsson JT, Agnarsson BA, Baker A, Sigurdsson A, Benediktsdottir KR, Jakobsdottir M, Xu J, Blondal T, Kostic J, Sun J, Ghosh S, Stacey SN, Mouy M, Saemundsdottir J, Backman VM, Kristjansson K, Tres A, Partin AW, Albers-Akkers MT, Godino-Ivan Marcos J, Walsh PC, Swinkels DW, Navarrete S (2007). Genome-wide association study identifies a second prostate cancer susceptibility variant at 8q24. Nat Genet.

[B6] Haiman CA, Patterson N, Freedman ML, Myers SR, Pike MC, Waliszewska A, Neubauer J, Tandon A, Schirmer C, McDonald GJ, Greenway SC, Stram DO, Le Marchand L, Kolonel LN, Frasco M, Wong D, Pooler LC, Ardlie K, Oakley-Girvan I, Whittemore AS, Cooney KA, John EM, Ingles SA, Altshuler D, Henderson BE, Reich D (2007). Multiple regions within 8q24 independently affect risk for prostate cancer. Nat Genet.

[B7] Yeager M, Orr N, Hayes RB, Jacobs KB, Kraft P, Wacholder S, Minichiello MJ, Fearnhead P, Yu K, Chatterjee N, Wang Z, Welch R, Staats BJ, Calle EE, Feigelson HS, Thun MJ, Rodriguez C, Albanes D, Virtamo J, Weinstein S, Schumacher FR, Giovannucci E, Willett WC, Cancel-Tassin G, Cussenot O, Valeri A, Andriole GL, Gelmann EP, Tucker M, Gerhard DS (2007). Genome-wide association study of prostate cancer identifies a second risk locus at 8q24. Nat Genet.

[B8] Tomlinson I, Webb E, Carvajal-Carmona L, Broderick P, Kemp Z, Spain S, Penegar S, Chandler I, Gorman M, Wood W, Barclay E, Lubbe S, Martin L, Sellick G, Jaeger E, Hubner R, Wild R, Rowan A, Fielding S, Howarth K, Silver A, Atkin W, Muir K, Logan R, Kerr D, Johnstone E, Sieber O, Gray R, Thomas H, Peto J (2007). A genome-wide association scan of tag SNPs identifies a susceptibility variant for colorectal cancer at 8q24.21. Nat Genet.

[B9] Zanke BW, Greenwood CMT, Rangrej J, Kustra R, Tenesa A, Farrington SM, Prendergast J, Olschwang S, Chiang T, Crowdy E, Ferretti V, Laflamme P, Sundararajan S, Roumy S, Olivier J-F, Robidoux F, Sladek R, Montpetit A, Campbell P, Bezieau S, O'Shea AM, Zogopoulos G, Cotterchio M, Newcomb P, McLaughlin J, Younghusband B, Green R, Green J, Porteous MEM, Campbell H (2007). Genome-wide association scan identifies a colorectal cancer susceptibility locus on chromosome 8q24. Nat Genet.

[B10] Witte JS (2007). Multiple prostate cancer risk variants on 8q24. Nat Genet.

[B11] Freedman ML, Haiman CA, Patterson N, McDonald GJ, Tandon A, Waliszewska A, Penney K, Steen RG, Ardlie K, John EM, Oakley-Girvan I, Whittemore AS, Cooney KA, Ingles SA, Altshuler D, Henderson BE, Reich D (2006). Admixture mapping identifies 8q24 as a prostate cancer risk locus in African-American men. Proc Natl Acad Sci USA.

[B12] Amundadottir LT, Sulem P, Gudmundsson J, Helgason A, Baker A, Agnarsson BA, Sigurdsson A, Benediktsdottir KR, Cazier JB, Sainz J, Jakobsdottir M, Kostic J, Magnusdottir DN, Ghosh S, Agnarsson K, Birgisdottir B, Le Roux L, Olafsdottir A, Blondal T, Andresdottir M, Gretarsdottir OS, Bergthorsson JT, Gudbjartsson D, Gylfason A, Thorleifsson G, Manolescu A, Kristjansson K, Geirsson G, Isaksson H, Douglas J (2006). A common variant associated with prostate cancer in European and African populations. Nat Genet.

[B13] Hunter DJ, Thomas G, Hoover RN, Chanock SJ (2007). Scanning the horizon: what is the future of genome-wide association studies in accelerating discoveries in cancer etiology and prevention?. Cancer Causes Control.

[B14] Chanock SJ, Manolio T, Boehnke M, Boerwinkle E, Hunter DJ, Thomas G, Hirschhorn JN, Abecasis G, Altshuler D, Bailey-Wilson JE, Brooks LD, Cardon LR, Daly M, Donnelly P, Fraumeni JF, Freimer NB, Gerhard DS, Gunter C, Guttmacher AE, Guyer MS, Harris EL, Hoh J, Hoover R, Kong CA, Merikangas KR, Morton CC, Palmer LJ, Phimister EG, Rice JP, Roberts J (2007). Replicating genotype-phenotype associations. Nature.

[B15] Liu ET (2005). Systems biology, integrative biology, predictive biology. Cell.

[B16] Kitano H (2002). Systems biology: a brief overview. Science.

[B17] Vidal M (2005). Interactome modeling. FEBS Lett.

[B18] Barabasi AL, Oltvai ZN (2004). Network biology: understanding the cell's functional organization. Nat Rev Genet.

[B19] Loscalzo J, Kohane I, Barabasi AL (2007). Human disease classification in the postgenomic era: a complex systems approach to human pathobiology. Mol Syst Biol.

[B20] Wang K, Li M, Bucan M (2007). Pathway-based approaches for analysis of genomewide association studies. Am J Hum Genet.

[B21] Durinck S, Moreau Y, Kasprzyk A, Davis S, De Moor B, Brazma A, Huber W (2005). BioMart and Bioconductor: a powerful link between biological databases and microarray data analysis. Bioinformatics.

[B22] Ashburner M, Ball CA, Blake JA, Botstein D, Butler H, Cherry JM, Davis AP, Dolinski K, Dwight SS, Eppig JT, Harris MA, Hill DP, Issel-Tarver L, Kasarskis A, Lewis S, Matese JC, Richardson JE, Ringwald M, Rubin GM, Sherlock G (2000). Gene ontology: tool for the unification of biology. The Gene Ontology Consortium. Nat Genet.

[B23] Al-Shahrour F, Arbiza L, Dopazo H, Huerta-Cepas J, Minguez P, Montaner D, Dopazo J (2007). From genes to functional classes in the study of biological systems. BMC Bioinformatics.

[B24] Al-Shahrour F, Diaz-Uriarte R, Dopazo J (2005). Discovering molecular functions significantly related to phenotypes by combining gene expression data and biological information. Bioinformatics.

[B25] R Development Core Team (2005). R: A language and environment for statistical computing. ISBN.

[B26] Benjamini Y, Hochberg Y (1995). Controlling the false discovery rate: a practical and powerful approach to multiple testing. Journal of the Royal Statistical Society Series B-Statistical Methodology.

[B27] Pollard KS, Dudoit S, Laan MJ van der (2004). Multiple testing procedures: R *multtest *package and applications to genomics.

[B28] Subramanian A, Kuehn H, Gould J, Tamayo P, Mesirov JP (2007). GSEA-P: a desktop application for Gene Set Enrichment Analysis. Bioinformatics.

[B29] Richardson AL, Wang ZC, De Nicolo A, Lu X, Brown M, Miron A, Liao X, Iglehart JD, Livingston DM, Ganesan S (2006). X chromosomal abnormalities in basal-like human breast cancer. Cancer Cell.

[B30] Chin K, DeVries S, Fridlyand J, Spellman PT, Roydasgupta R, Kuo WL, Lapuk A, Neve RM, Qian Z, Ryder T, Chen F, Feiler H, Tokuyasu T, Kingsley C, Dairkee S, Meng Z, Chew K, Pinkel D, Jain A, Ljung BM, Esserman L, Albertson DG, Waldman FM, Gray JW (2006). Genomic and transcriptional aberrations linked to breast cancer pathophysiologies. Cancer Cell.

[B31] Chang HY, Nuyten DS, Sneddon JB, Hastie T, Tibshirani R, Sorlie T, Dai H, He YD, van't Veer LJ, Bartelink H, Rijn M van de, Brown PO, Vijver MJ van de (2005). Robustness, scalability, and integration of a wound-response gene expression signature in predicting breast cancer survival. Proc Natl Acad Sci USA.

[B32] Furuta S, Wang JM, Wei S, Jeng YM, Jiang X, Gu B, Chen PL, Lee EY, Lee WH (2006). Removal of BRCA1/CtIP/ZBRK1 repressor complex on ANG1 promoter leads to accelerated mammary tumor growth contributed by prominent vasculature. Cancer Cell.

[B33] van 't Veer LJ, Dai H, Vijver MJ van de, He YD, Hart AA, Mao M, Peterse HL, Kooy K van der, Marton MJ, Witteveen AT, Schreiber GJ, Kerkhoven RM, Roberts C, Linsley PS, Bernards R, Friend SH (2002). Gene expression profiling predicts clinical outcome of breast cancer. Nature.

[B34] Lee S, Medina D, Tsimelzon A, Mohsin SK, Mao S, Wu Y, Allred DC (2007). Alterations of gene expression in the development of early hyperplastic precursors of breast cancer. Am J Pathol.

[B35] Rual JF, Venkatesan K, Hao T, Hirozane-Kishikawa T, Dricot A, Li N, Berriz GF, Gibbons FD, Dreze M, Ayivi-Guedehoussou N, Klitgord N, Simon C, Boxem M, Milstein S, Rosenberg J, Goldberg DS, Zhang LV, Wong SL, Franklin G, Li S, Albala JS, Lim J, Fraughton C, Llamosas E, Cevik S, Bex C, Lamesch P, Sikorski RS, Vandenhaute J, Zoghbi HY (2005). Towards a proteome-scale map of the human protein-protein interaction network. Nature.

[B36] Stelzl U, Worm U, Lalowski M, Haenig C, Brembeck FH, Goehler H, Stroedicke M, Zenkner M, Schoenherr A, Koeppen S, Timm J, Mintzlaff S, Abraham C, Bock N, Kietzmann S, Goedde A, Toksoz E, Droege A, Krobitsch S, Korn B, Birchmeier W, Lehrach H, Wanker EE (2005). A human protein-protein interaction network: a resource for annotating the proteome. Cell.

[B37] Freeman LC (1977). A set of measures of centrality based on betweenness. Sociometry.

[B38] Dixon AL, Liang L, Moffatt MF, Chen W, Heath S, Wong KC, Taylor J, Burnett E, Gut I, Farrall M, Lathrop GM, Abecasis GR, Cookson WO (2007). A genome-wide association study of global gene expression. Nat Genet.

[B39] Shannon P, Markiel A, Ozier O, Baliga NS, Wang JT, Ramage D, Amin N, Schwikowski B, Ideker T (2003). Cytoscape: a software environment for integrated models of biomolecular interaction networks. Genome Res.

[B40] Subramanian A, Tamayo P, Mootha VK, Mukherjee S, Ebert BL, Gillette MA, Paulovich A, Pomeroy SL, Golub TR, Lander ES, Mesirov JP (2005). Gene set enrichment analysis: a knowledge-based approach for interpreting genome-wide expression profiles. Proc Natl Acad Sci USA.

[B41] Cox DG, Hankinson SE, Hunter DJ (2006). Polymorphisms of the *AURKA *(*STK15/Aurora Kinase*) gene and breast cancer risk (United States). Cancer Causes Control.

[B42] Ewart-Toland A, Dai Q, Gao YT, Nagase H, Dunlop MG, Farrington SM, Barnetson RA, Anton-Culver H, Peel D, Ziogas A, Lin D, Miao X, Sun T, Ostrander EA, Stanford JL, Langlois M, Chan JM, Yuan J, Harris CC, Bowman ED, Clayman GL, Lippman SM, Lee JJ, Zheng W, Balmain A (2005). *Aurora-A/STK15 *T+91A is a general low penetrance cancer susceptibility gene: a meta-analysis of multiple cancer types. Carcinogenesis.

[B43] Lo YL, Yu JC, Chen ST, Yang HC, Fann CS, Mau YC, Shen CY (2005). Breast cancer risk associated with genotypic polymorphism of the mitosis-regulating gene *Aurora-A/STK15/BTAK*. Int J Cancer.

[B44] Sun T, Miao X, Wang J, Tan W, Zhou Y, Yu C, Lin D (2004). Functional Phe31Ile polymorphism in Aurora A and risk of breast carcinoma. Carcinogenesis.

[B45] Ewart-Toland A, Briassouli P, de Koning JP, Mao JH, Yuan J, Chan F, MacCarthy-Morrogh L, Ponder BA, Nagase H, Burn J, Ball S, Almeida M, Linardopoulos S, Balmain A (2003). Identification of Stk6/STK15 as a candidate low-penetrance tumor-susceptibility gene in mouse and human. Nat Genet.

[B46] Cox A, Dunning AM, Garcia-Closas M, Balasubramanian S, Reed MW, Pooley KA, Scollen S, Baynes C, Ponder BA, Chanock S, Lissowska J, Brinton L, Peplonska B, Southey MC, Hopper JL, McCredie MR, Giles GG, Fletcher O, Johnson N, dos Santos Silva I, Gibson L, Bojesen SE, Nordestgaard BG, Axelsson CK, Torres D, Hamann U, Justenhoven C, Brauch H, Chang-Claude J, Kropp S (2007). A common coding variant in CASP8 is associated with breast cancer risk. Nat Genet.

[B47] Song B, Margolin S, Skoglund J, Zhou X, Rantala J, Picelli S, Werelius B, Lindblom A (2007). TGFBR1(*)6A and Int7G24A variants of transforming growth factor-beta receptor 1 in Swedish familial and sporadic breast cancer. Br J Cancer.

[B48] Cox DG, Penney K, Guo Q, Hankinson SE, Hunter DJ (2007). TGFB1 and TGFBR1 polymorphisms and breast cancer risk in the Nurses' Health Study. BMC Cancer.

[B49] Chen T, Jackson CR, Link A, Markey MP, Colligan BM, Douglass LE, Pemberton JO, Deddens JA, Graff JR, Carter JH (2006). Int7G24A variant of transforming growth factor-beta receptor type I is associated with invasive breast cancer. Clin Cancer Res.

[B50] Kaklamani VG, Baddi L, Liu J, Rosman D, Phukan S, Bradley C, Hegarty C, McDaniel B, Rademaker A, Oddoux C, Ostrer H, Michel LS, Huang H, Chen Y, Ahsan H, Offit K, Pasche B (2005). Combined genetic assessment of transforming growth factor-beta signaling pathway variants may predict breast cancer risk. Cancer Res.

[B51] Kaklamani VG, Hou N, Bian Y, Reich J, Offit K, Michel LS, Rubinstein WS, Rademaker A, Pasche B (2003). TGFBR1*6A and cancer risk: a meta-analysis of seven case-control studies. J Clin Oncol.

[B52] Perou CM, Sorlie T, Eisen MB, Rijn M van de, Jeffrey SS, Rees CA, Pollack JR, Ross DT, Johnsen H, Akslen LA, Fluge O, Pergamenschikov A, Williams C, Zhu SX, Lonning PE, Borresen-Dale AL, Brown PO, Botstein D (2000). Molecular portraits of human breast tumours. Nature.

[B53] Pasche B (2008). Recent advances in breast cancer genetics. Cancer Treat Res.

[B54] Vijver MJ van de, He YD, van't Veer LJ, Dai H, Hart AA, Voskuil DW, Schreiber GJ, Peterse JL, Roberts C, Marton MJ, Parrish M, Atsma D, Witteveen A, Glas A, Delahaye L, Velde T van der, Bartelink H, Rodenhuis S, Rutgers ET, Friend SH, Bernards R (2002). A gene-expression signature as a predictor of survival in breast cancer. N Engl J Med.

[B55] Furuta S, Jiang X, Gu B, Cheng E, Chen PL, Lee WH (2005). Depletion of BRCA1 impairs differentiation but enhances proliferation of mammary epithelial cells. Proc Natl Acad Sci USA.

[B56] Walker LC, Waddell N, Ten Haaf A, Grimmond S, Spurdle AB (2007). Use of expression data and the CGEMS genome-wide breast cancer association study to identify genes that may modify risk in BRCA1/2 mutation carriers. Breast Cancer Res Treat.

[B57] Pujana MA, Han JD, Starita LM, Stevens KN, Tewari M, Ahn JS, Rennert G, Moreno V, Kirchhoff T, Gold B, Assmann V, Elshamy WM, Rual JF, Levine D, Rozek LS, Gelman RS, Gunsalus KC, Greenberg RA, Sobhian B, Bertin N, Venkatesan K, Ayivi-Guedehoussou N, Sole X, Hernandez P, Lazaro C, Nathanson KL, Weber BL, Cusick ME, Hill DE, Offit K (2007). Network modeling links breast cancer susceptibility and centrosome dysfunction. Nat Genet.

[B58] Chang JT, Nevins JR (2006). GATHER: a systems approach to interpreting genomic signatures. Bioinformatics.

[B59] Wennmalm K, Miller LD, Bergh J (2007). A gene signature in breast cancer. N Engl J Med.

[B60] Yu JX, Sieuwerts AM, Zhang Y, Martens JW, Smid M, Klijn JG, Wang Y, Foekens JA (2007). Pathway analysis of gene signatures predicting metastasis of node-negative primary breast cancer. BMC Cancer.

[B61] Shen R, Ghosh D, Chinnaiyan AM (2004). Prognostic meta-signature of breast cancer developed by two-stage mixture modeling of microarray data. BMC Genomics.

[B62] Zhang Z, Chen D, Fenstermacher DA (2007). Integrated analysis of independent gene expression microarray datasets improves the predictability of breast cancer outcome. BMC Genomics.

[B63] Vuaroqueaux V, Urban P, Labuhn M, Delorenzi M, Wirapati P, Benz CC, Flury R, Dieterich H, Spyratos F, Eppenberger U, Eppenberger-Castori S (2007). Low *E2F1 *transcript levels are a strong determinant of favorable breast cancer outcome. Breast Cancer Res.

[B64] Untergasser G, Steurer M, Zimmermann M, Hermann M, Kern J, Amberger A, Gastl G, Gunsilius E (2008). The Dickkopf-homolog 3 is expressed in tumor endothelial cells and supports capillary formation. Int J Cancer.

[B65] Guo H, Lin Y, Zhang H, Liu J, Zhang N, Li Y, Kong D, Tang Q, Ma D (2007). Tissue factor pathway inhibitor-2 was repressed by CpG hypermethylation through inhibition of KLF6 binding in highly invasive breast cancer cells. BMC Mol Biol.

[B66] Renehan AG, Harvie M, Howell A (2006). Insulin-like growth factor (IGF)-I, IGF binding protein-3, and breast cancer risk: eight years on. Endocr Relat Cancer.

[B67] Bachmann HS, Otterbach F, Callies R, Nuckel H, Bau M, Schmid KW, Siffert W, Kimmig R (2007). The AA genotype of the regulatory *BCL2 *promoter polymorphism (938C>A) is associated with a favorable outcome in lymph node negative invasive breast cancer patients. Clin Cancer Res.

[B68] Hassan S, Baccarelli A, Salvucci O, Basik M (2008). Plasma stromal cell-derived factor-1: host derived marker predictive of distant metastasis in breast cancer. Clin Cancer Res.

[B69] Hsu EL, Chen N, Westbrook A, Wang F, Zhang R, Taylor RT, Hankinson O (2008). CXCR4 and CXCL12 down-regulation: A novel mechanism for the chemoprotection of 3,3'-diindolylmethane for breast and ovarian cancers. Cancer Lett.

[B70] Wendt MK, Cooper AN, Dwinell MB (2008). Epigenetic silencing of *CXCL12 *increases the metastatic potential of mammary carcinoma cells. Oncogene.

[B71] Pupa SM, Argraves WS, Forti S, Casalini P, Berno V, Agresti R, Aiello P, Invernizzi A, Baldassari P, Twal WO, Mortarini R, Anichini A, Menard S (2004). Immunological and pathobiological roles of fibulin-1 in breast cancer. Oncogene.

[B72] Greene LM, Twal WO, Duffy MJ, McDermott EW, Hill AD, O'Higgins NJ, McCann AH, Dervan PA, Argraves WS, Gallagher WM (2003). Elevated expression and altered processing of fibulin-1 protein in human breast cancer. Br J Cancer.

[B73] Unoki M, Nakamura Y (2001). Growth-suppressive effects of *BPOZ *and *EGR2*, two genes involved in the *PTEN *signaling pathway. Oncogene.

[B74] Carvalho I, Milanezi F, Martins A, Reis RM, Schmitt F (2005). Overexpression of platelet-derived growth factor receptor alpha in breast cancer is associated with tumour progression. Breast Cancer Res.

[B75] Lerebours F, Olschwang S, Thuille B, Schmitz A, Fouchet P, Buecher B, Martinet N, Galateau F, Thomas G (1999). Fine deletion mapping of chromosome 8p in non-small-cell lung carcinoma. Int J Cancer.

[B76] Komiya A, Suzuki H, Ueda T, Aida S, Ito N, Shiraishi T, Yatani R, Emi M, Yasuda K, Shimazaki J, Ito H (1997). *PRLTS *gene alterations in human prostate cancer. Jpn J Cancer Res.

[B77] Seitz S, Werner S, Fischer J, Nothnagel A, Schlag PM, Scherneck S (2000). Refined deletion mapping in sporadic breast cancer at chromosomal region 8p12-p21 and association with clinicopathological parameters. Eur J Cancer.

[B78] Yaremko ML, Kutza C, Lyzak J, Mick R, Recant WM, Westbrook CA (1996). Loss of heterozygosity from the short arm of chromosome 8 is associated with invasive behavior in breast cancer. Genes Chromosomes Cancer.

[B79] Rennstam K, Ahlstedt-Soini M, Baldetorp B, Bendahl PO, Borg A, Karhu R, Tanner M, Tirkkonen M, Isola J (2003). Patterns of chromosomal imbalances defines subgroups of breast cancer with distinct clinical features and prognosis. A study of 305 tumors by comparative genomic hybridization. Cancer Res.

[B80] Xu M, Kao MC, Nunez-Iglesias J, Nevins JR, West M, Zhou XJ (2008). An integrative approach to characterize disease-specific pathways and their coordination: a case study in cancer. BMC Genomics.

[B81] Forbes S, Clements J, Dawson E, Bamford S, Webb T, Dogan A, Flanagan A, Teague J, Wooster R, Futreal PA, Stratton MR (2006). Cosmic 2005. Br J Cancer.

[B82] Kitano H (2004). Cancer as a robust system: implications for anticancer therapy. Nat Rev Cancer.

[B83] Frazer KA, Ballinger DG, Cox DR, Hinds DA, Stuve LL, Gibbs RA, Belmont JW, Boudreau A, Hardenbol P, Leal SM, Pasternak S, Wheeler DA, Willis TD, Yu F, Yang H, Zeng C, Gao Y, Hu H, Hu W, Li C, Lin W, Liu S, Pan H, Tang X, Wang J, Wang W, Yu J, Zhang B, Zhang Q, Zhao H (2007). A second generation human haplotype map of over 3.1 million SNPs. Nature.

[B84] Hanahan D, Weinberg RA (2000). The hallmarks of cancer. Cell.

[B85] Eswarakumar VP, Lax I, Schlessinger J (2005). Cellular signaling by fibroblast growth factor receptors. Cytokine Growth Factor Rev.

[B86] Cuevas BD, Winter-Vann AM, Johnson NL, Johnson GL (2006). MEKK1 controls matrix degradation and tumor cell dissemination during metastasis of polyoma middle-T driven mammary cancer. Oncogene.

[B87] Antoniou AC, Spurdle AB, Sinilnikova OM, Healey S, Pooley KA, Schmutzler RK, Versmold B, Engel C, Meindl A, Arnold N, Hofmann W, Sutter C, Niederacher D, Deissler H, Caldes T, Kampjarvi K, Nevanlinna H, Simard J, Beesley J, Chen X, Neuhausen SL, Rebbeck TR, Wagner T, Lynch HT, Isaacs C, Weitzel J, Ganz PA, Daly MB, Tomlinson G, Olopade OI (2008). Common breast cancer-predisposition alleles are associated with breast cancer risk in BRCA1 and BRCA2 mutation carriers. Am J Hum Genet.

[B88] Kanehisa M, Goto S, Hattori M, Aoki-Kinoshita KF, Itoh M, Kawashima S, Katayama T, Araki M, Hirakawa M (2006). From genomics to chemical genomics: new developments in KEGG. Nucleic Acids Res.

[B89] Garraway LA, Widlund HR, Rubin MA, Getz G, Berger AJ, Ramaswamy S, Beroukhim R, Milner DA, Granter SR, Du J, Lee C, Wagner SN, Li C, Golub TR, Rimm DL, Meyerson ML, Fisher DE, Sellers WR (2005). Integrative genomic analyses identify *MITF *as a lineage survival oncogene amplified in malignant melanoma. Nature.

