# Supplementary material for: Biological processes, properties and molecular wiring diagrams of candidate low-penetrance breast cancer susceptibility genes
Source: BMC Med Genomics. 2008 Dec 18;1:62. doi: 10.1186/1755-8794-1-62 (PMC2628924; doi:10.1186/1755-8794-1-62)
Supplement: Additional file 2 — Results of the GSEA algorithm. [file 1755-8794-1-62-S2.pdf]

Additional file 2. Results of the application of the GSEA algorithm to breast cancer WGAS ordered gene lists (only GO terms with FDR *P* values < 0.05 are shown)*

GO accession	Term	FDR *P* value
**WGAS lowest *P* value per gene locus ($\log_2$ transformed; GSEA weighting exponent p = 1)**		
GO:0007155	Cell Adhesion	0.001
**WGAS (gene index order; GSEA weighting exponent p = 0)**		
GO:0007154	Cell Communication	< 0.001
GO:0007155	Cell Adhesion	< 0.001
GO:0006810	Transport	< 0.001
GO:0007275	Multicellular Organismal Development	< 0.001
GO:0048856	Anatomical Structure Development	< 0.001
GO:0050794	Regulation of Cellular Process	< 0.001
GO:0048869	Cellular Developmental Process	0.003
GO:0050793	Regulation of Development	0.004
GO:0044238	Primary Metabolic Process	0.004
GO:0065008	Regulation of Biological Quality	0.005
GO:0044237	Cellular Metabolic Process	0.006
GO:0065009	Regulation of Molecular Function	0.011
GO:0051094	Positive Regulation of Developmental Process	0.018
GO:0009056	Catabolic Process	0.030
GO:0009653	Anatomical Structure Morphogenesis	0.032
GO:0032879	Regulation of Localization	0.035
GO:0048468	Cell Development	0.036
GO:0016265	Death	0.041
GO:0006955	Immune Response	0.046
GO:0016043	Cellular Component Organization and Biogenesis	0.049

*In the WGAS OR/*P* value ordered list the top-ranked term was Cell Adhesion (FDR *P* value = 0.26)
