# Supplementary figures and images for: Biological processes, properties and molecular wiring diagrams of candidate low-penetrance breast cancer susceptibility genes
Source: BMC Med Genomics. 2008 Dec 18;1:62. doi: 10.1186/1755-8794-1-62 (PMC2628924; doi:10.1186/1755-8794-1-62)

## Luminal A

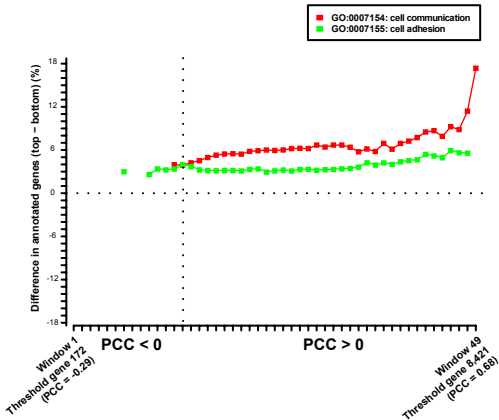

Supplement: Additional file 4 — Asymmetries in the rank of somatic copy number-gene expression correlations in luminal A tumors. [file 1755-8794-1-62-S4.pdf]
